# Glucose Variability: How Does It Work?

**DOI:** 10.3390/ijms22157783

**Published:** 2021-07-21

**Authors:** Vadim V. Klimontov, Olga V. Saik, Anton I. Korbut

**Affiliations:** 1 Laboratory of Endocrinology, Research Institute of Clinical and Experimental Lymphology—Branch of the Institute of Cytology and Genetics, Siberian Branch of Russian Academy of Sciences (RICEL—Branch of IC&G SB RAS), 630060 Novosibirsk, Russia; saik@bionet.nsc.ru (O.V.S.); korbutai@niikel.ru (A.I.K.); 2 Laboratory of Computer Proteomics, Federal Research Center Institute of Cytology and Genetics, Siberian Branch of the Russian Academy of Sciences (IC&G SB RAS), 630090 Novosibirsk, Russia

**Keywords:** diabetes, hyperglycemia, hypoglycemia, glucose variability, complications, gene, epigenetic, signal pathways

## Abstract

A growing body of evidence points to the role of glucose variability (GV) in the development of the microvascular and macrovascular complications of diabetes. In this review, we summarize data on GV-induced biochemical, cellular and molecular events involved in the pathogenesis of diabetic complications. Current data indicate that the deteriorating effect of GV on target organs can be realized through oxidative stress, glycation, chronic low-grade inflammation, endothelial dysfunction, platelet activation, impaired angiogenesis and renal fibrosis. The effects of GV on oxidative stress, inflammation, endothelial dysfunction and hypercoagulability could be aggravated by hypoglycemia, associated with high GV. Oscillating hyperglycemia contributes to beta cell dysfunction, which leads to a further increase in GV and completes the vicious circle. In cells, the GV-induced cytotoxic effect includes mitochondrial dysfunction, endoplasmic reticulum stress and disturbances in autophagic flux, which are accompanied by reduced viability, activation of apoptosis and abnormalities in cell proliferation. These effects are realized through the up- and down-regulation of a large number of genes and the activity of signaling pathways such as PI3K/Akt, NF-κB, MAPK (ERK), JNK and TGF-β/Smad. Epigenetic modifications mediate the postponed effects of glucose fluctuations. The multiple deteriorative effects of GV provide further support for considering it as a therapeutic target in diabetes.

## 1. Introduction

The concept of glucose variability (GV) is gaining increasing attention from scientists and clinicians. In recent decades, various methodological approaches have been developed to assess fluctuations in glucose levels [1,2]. Some GV metrics are implemented in the standardized analysis of continuous glucose monitoring (CGM) data [3]. The minimization of GV is recognized as a therapeutic target in diabetes management [4,5]. The growing attention to GV is explained primarily by the predictive value of this phenomenon. Many observational studies and post hoc analyses of randomized clinical trials have demonstrated that short-term GV and variability of glycated hemoglobin A1c (HbA1c) are associated with an increased risk of diabetic microvascular and macrovascular complications [6,7,8,9,10,11]. Some studies have also documented the association between high GV and mortality rates in patients with type 2 diabetes (T2D) [4,10,12]. Accumulating data indicate that ambient hyperglycemia can be even more dangerous for the cells of the cardiovascular and nervous systems, and renal and pancreatic beta cells than persistently high glucose levels [13,14,15,16,17,18]. The molecular pathways of the GV effect have been partially discovered in recent years and must be systematized.

In this review, we summarize data on GV-related biochemical/pathophysiological, cellular and molecular events in conditions of high GV, which may be important for the development of diabetic complications. We consequently searched the relevant articles in Pubmed/MEDLINE, Scopus and the Web of Science with the following keywords: “glucose variability” or “glycemic variability”, “glucose fluctuation” or “glycemic fluctuation”, “glucose excursion” or “glycemic excursion”, “glucose oscillations” or “glycemic oscillations”, “intermittent high glucose”, “fluctuating glucose’’. We also combined these terms with “hypoglycemia”. Reference lists of relevant reviews and articles were thoroughly checked to ensure all relevant studies were obtained. Both experimental and clinical studies were reviewed.

## 2. Biochemical and Pathophysiological Abnormalities Induced by Excessive Glucose Fluctuations

### 2.1. Oxidative Stress and Non-Enzymatic Glycation

It is generally accepted that hyperglycemia induces the overproduction of reactive oxygen species (ROS) and impairs the endogenous antioxidant defense, a condition known as oxidative stress. A number of experimental studies have indicated that intermittently high glucose (IHG) can generate even more severe oxidative stress than a constantly high glucose (CHG) level. Specifically, this effect was described in cultured endothelial cells [13,19,20,21], podocytes [22], adipocytes [23], Schwann cells [14,15] and pancreatic beta cells [24]. The effect of IHG was related to the enhanced activation of nicotinamide adenine dinucleotide phosphate (NADPH) oxidase [13] and included excessive ROS production, oxidative DNA damage and the depletion of superoxide dismutase activity [19,20]. An increase in plasma levels of malondialdehyde and 8-isoprostaglandin, well-known oxidative stress markers, as well as the enhanced generation of ROS in vascular endothelial cells, was demonstrated in rats with blood glucose fluctuations (5.5–20 mmol/L) induced by intermittent intravenous glucose infusion [25]. In patients with T2D, the levels of 8-iso-prostaglandin F2α, thiobarbituric acid-reactive substances and 8-hydroxydeoxyguanosine showed positive correlations with the mean amplitude of glucose excursions (MAGE), but not with the HbA1c level. Long-term GV, estimated by the standard deviation (SD) of HbA1c levels over a 2-year period, was also correlated with these oxidative stress markers [26]. Meanwhile, in other studies, no association between the urinary excretion of 8-iso-prostaglandin-F2α and CGM-derived GV parameters was revealed in patients with type 1 diabetes [27] and well-controlled T2D [28]. Recent research has indicated a correlation between 1,5-anhydroglucitol, an intermediate-term marker of GV, and ROS metabolites in patients with T2D and HbA1c below 8% [29].

A growing body of evidence indicates the role of GV as a predictor of hypoglycemia [10,30,31]. The alternation of hyperglycemic and hypoglycemic episodes, a characteristic feature of increased GV, can be a powerful inducer of oxidative stress. It was demonstrated that an episode of 2 h of hyperglycemia, followed by the recovery from the induced 2 h of hypoglycemia, aggravates oxidative stress in both healthy subjects and patients with type 1 diabetes (T1D) [32,33].

In diabetic conditions, hyperglycemia promotes the accumulation of advanced glycation end-products (AGEs). The activation of AGE receptors can lead to oxidative stress, low-grade inflammation and other events contributing to vascular complications [34]. However, little is known about the role of GV in the formation of AGEs. It was demonstrated that, among subjects with prediabetes, the levels of nitrotyrosine, a marker of nitrosative stress, and glyceraldehyde-derived AGEs were higher in those with increased MAGE [35,36]. Recent data indicate that acute glucose fluctuations up-regulate the expression of the receptor for AGEs in rat podocytes [37].

At present, oxidative stress and non-enzymatic glycation are considered as the mechanisms of “metabolic memory”, which determine the extent of the effect of glycemic control on metabolism and clinical outcomes in diabetes [35,36]. It was shown that the increased production of ROS in cultured human endothelial cells, caused by an excess of glucose (30 mmol/L), persists for at least a week after the normalization of glucose levels [38]. The incubation of human umbilical vein endothelial cells (HUVECs) in conditions of CHG (25 mmol/L) or IHG (24 h in 5 mmol/L, followed by 24 h in 25 mmol/L) for 2 weeks resulted in the up-regulation of ROS production. The generation of ROS was increased a week after the normalization of glucose levels, especially in cells that were previously incubated with IHG. Therefore, the memory effect can be more pronounced in high-GV conditions [37].

### 2.2. Chronic Low-Grade Inflammation

The activation of inflammatory pathways plays an important role in the pathogenesis of diabetic complications [39,40]. Increased GV contributes to the inflammatory response. As compared to CHG, oscillating glucose was a more potent inducer of intercellular adhesion molecule 1 (ICAM-1), vascular cell adhesion molecule 1 (VCAM-1), E-selectin and interleukin 6 (IL-6) expression in HUVECs. This effect was related to the oxidative stress and activation of poly (ADP-ribose) polymerase and protein kinase C [41,42]. The exposure to IHG enhanced the secretion of IL-6 and tumor necrosis factor α (TNF-α), the inflammatory cytokines, by activated monocytes; this effect was partly attributable to the inherent osmotic stress [43]. The expression and secretion of interleukin 18 (IL-18) in mouse peritoneal macrophages were increased to a greater extent under the influence of IHG than CHG; this effect was mediated by the c-Jun N-terminal kinase (JNK) signaling pathway [44]. In rat podocytes, acute glucose fluctuations induced the expression of TNF-α and interleukin 1 beta (IL-1β) to a greater extent than CHG [22]. In adipocytes, IHG induced a greater increase in the expression and secretion of IL-18 and monocyte chemoattractant protein 1 (MCP-1) than CHG [23]. An inflammatory response to fluctuating glucose has also been demonstrated in vivo. In rats, blood glucose fluctuations induced by intermittent glucose infusions increased the expression of IL-6, TNF-α and ICAM-1 in vascular endothelial cells [25].

Hypoglycemia may act as an additional trigger of inflammation under oscillating conditions. In cultured macrophages, the intermittent episodes of hypoglycemia and hyperglycemia (3–15 mmol/L) promoted M1 polarization and an inflammatory response, estimated by the secretion of integrin alpha X, IL-1β, TNF-α, IL-6 and MCP-1, via a mechanism involving the Toll-like receptor 4 (TLR4)–interferon regulatory factor 5 (IRF5) pathway [45]. It was found that hypoglycemia promotes the mobilization of specific leukocyte subsets into the bloodstream and induces proinflammatory changes in the leukocytes in healthy individuals and patients with T1D [46]. Specifically, the mobilization of cluster of differentiation 8-positive (CD8+) T cells, cytotoxic natural killers and natural killer T cells, as well as non-classical monocytes, was observed [47]. In patients with T1D, an episode of two-hour hypoglycemia was followed by an increase in the levels of soluble ICAM-1 (sICAM-1) and IL-6 [48]. High blood glucose, replacing hypoglycemia, caused a further increase in the concentrations of sICAM-1 and IL-6 [49]. In T1D patients on pump therapy, the number of hypoglycemic episodes predicted plasma levels of ICAM-1, VCAM-1 and E-selectin [50]. In subjects with T1D, acute hyperglycemia was followed by an elevation in urinary excretion of a number of proinflammatory chemokines and cytokines [51]. In non-diabetic subjects with reactive hypoglycemia, Eik W. et al. observed a rise in the plasma levels of proinflammatory (IL-2, IL-5 and IL-17) and anti-inflammatory cytokines (IL-4, IL-1RA, IL-2R, IL-13 and fibroblast growth factor basic) during hypoglycemia after the glucose load [52]. An increase in adrenaline mediates the inflammatory response associated with hypoglycemia in non-diabetic subjects and patients with T1D [53].

Associations between inflammatory markers and GV have been observed in some clinical studies. A correlation between high-sensitivity C-reactive protein (hsCRP) levels and CGM-derived SD was reported in adolescents with T1D [54]. In patients with T2D, hsCRP correlated with both short-term (MAGE index) and long-term GV (SD of HbA1c level over two years) [26]. In another study, acid α_1_-glycoprotein, but not hsCRP, was related to GV indices reflecting hyperglycemic fluctuations in subjects with T2D [55]. An association between the coefficient of variation (CV), calculated from CGM data, and blood IL-6 levels was shown in non-diabetic persons with metabolic syndrome [56].

### 2.3. Endothelial Dysfunction and Vascular Remodeling

Endothelial dysfunction is considered as an important player in the pathogenesis of diabetic vascular complications. Hyperglycemia impairs the vascular endothelium function through the polyol and hexosamine pathways, protein kinase C (PKC) activation and generation of AGEs, all of which lead to ROS overproduction, the dysregulation of growth factors and cytokines and epigenetic changes [57]. A growing body of evidence indicates a deleterious effect of supraphysiological glucose fluctuations on endothelial function. When compared to CHG, IHG produced a stronger impairing effect on NO synthesis in cultured HUVECs [58]. Increased GV, even in the absence of high glucose levels, can suppress the endothelial defense against hyperglycemia-induced metabolic disorders. Modeling short-term fluctuations similar to those in diabetic patients changed the synthesis of a number of key enzymes in cultured human endothelial cells. Specifically, a decrease in the expression of superoxide dismutase 2, heme oxygenase 1, glyoxalase and transketolase was observed [59]. It was demonstrated that IHG can promote vascular endothelial senescence to a greater extent than CHG, which is partially dependent on oxidative stress [60].

Horvath et al. compared the effect of stable and intermittent hyperglycemia on endothelial function in rats with streptozotocin-induced diabetes. The endothelium-dependent dilation was significantly impaired in rats that were periodically injected with insulin compared with animals that did not receive treatment, despite the lower mean blood glucose levels in the insulin-treated group [61]. In patients with T1D, glucose fluctuations in the range of 5–15 mmol/L induced a more severe impairment of endothelium-dependent arterial dilation compared to that induced by stable hyperglycemia (10 and 15 mmol/L). The effect of intermittent hyperglycemia on endothelial function has been associated with oxidative stress [49]. In non-complicated T2D subjects receiving a diet and/or metformin, mean postprandial glucose excursions correlated negatively with flow-mediated arterial dilation [62]. Similarly, enhanced GV was related to flow-mediated dilation in patients with T2D and coronary artery disease [44]. In children with T1D, flow-mediated dilation was related to hypoglycemia, but not MAGE or other GV metrics [63]. It was found that endothelial microparticles, a novel surrogate marker of endothelial injury and dysfunction, are differentially produced in response to hypoglycemia in subjects with and without T2D. Insulin-induced hypoglycemia provoked a more dramatic increase in the levels of CD31+ and CD105+ endothelial microparticles in individuals with T2D compared to controls [64].

There are some data indicating endothelial dysfunction in individuals with impaired glucose tolerance, which can be considered as an equivalent to enhanced GV in non-diabetic subjects. It was reported that plasma levels of von Willebrand factor, and soluble E-selectin, two widely used markers of endothelial damage, are elevated in patients with impaired glucose tolerance [65]. In the population-based Maastricht Study, which enrolled 2758 participants, the glucose peak during a glucose tolerance test was independently associated with aortic stiffness and carotid remodeling, as well as with microvascular function, estimated by retinal arteriolar dilation and heat-induced skin hyperemia [66]. In subjects with T2D and unstable angina, the SD of blood glucose was an independent predictor for coronary artery calcification [67].

### 2.4. Platelet Activation and Hypercoagulability

The interactions between activated vascular cells and vulnerable atheromatous plaques are considered as a cornerstone in atherothrombotic burden in diabetes [68]. Some data indicate that enhanced GV could be related to platelet reactivity. Specifically, in patients with T2D, postprandial hyperglycemia was associated with platelet activation, estimated by the urinary excretion of 11-dehydro-thromboxane B2. The excretion rate was reduced by the treatment with acarbose, following earlier decreases in postprandial glucose and MAGE [69]. In subjects with well-controlled T2D via clopidogrel therapy, MAGE and CV provided independent and additional diagnostic significance in identifying patients with high platelet reactivity [70]. At the same time, no impact of acute glucose load (75 g) on platelet aggregation was observed in patients with T2D or acute coronary syndrome [71].

While the effect of hyperglycemic fluctuations on platelets warrants further research, the role of hypoglycemia in platelet activation and hypercoagulability is well established. In a study with hyperinsulinemic-hypoglycemic and euglycemic clamps, hypoglycemia mobilized monocytes, increased platelet reactivity and promoted the interaction between platelets and proinflammatory monocytes in healthy subjects [72]. In the study of Ceriello et al., hypoglycemia increased plasma levels of prothrombin fragment 1 + 2, thrombin-antithrombin III complexes and plasminogen activator inhibitor-1 (PAI-1) in both healthy subjects and people with diabetes. The transition from hypoglycemia to normoglycemia was accompanied by a significant improvement in coagulation parameters. On the contrary, hyperglycemia following hypoglycemia worsened coagulation markers; the effect persisted even after an additional 6 h of normoglycemia [33].

### 2.5. Impaired Angiogenesis

An angiogenic paradox has been described in diabetes, which refers to the excessive angiogenesis in retinopathy and nephropathy and suppression of blood vessel growth in limb and myocardial ischemia [73]. It was found that, according to this pattern, GV causes a bidirectional effect on angiogenesis. Acute glucose fluctuations (in the range of 5–25 mmol/L) impaired the proliferation of HUVECs and angiogenesis in vitro and delayed wound healing in mice. The effect of IHG on angiogenesis was more prominent than that of CHG [74]. In agreement with these data, the modeling of increased GV in mice impaired ischemia-induced angiogenesis in the hind limb by the suppression of vascular endothelial growth factor (VEGF) production [75]. At the same time, both CHG and IHG up-regulated VEGF in human retinal endothelial cells. The IHG effect on cell proliferation and VEGF expression was mediated via mitochondrial ROS overproduction [21].

The dysfunction and count abnormalities of endothelial progenitor cells (EPCs), which are derived from the bone marrow and involved in endothelial repair and new blood vessel formation, have been observed in diabetes [76]. The direct influence of IHG on EPCs has not been tested. Nonetheless, in patients with the T1D, the J-index, a GV parameter, correlated negatively with CD34^+^ EPC count [77]. In turn, reducing GV with continuous subcutaneous insulin infusion increased the EPC levels in subjects with T1D [78]. In patients with T1D, the levels of hematopoietic stem/progenitor cells (CD34^+^ CD133^+^, CD34^+^ CD45^dim^) were reduced and correlated positively with CV and time in hypoglycemia estimated by flash glucose monitoring; the relationships were mitigated in long-lasting diabetes [79].

### 2.6. Renal Fibrosis

More than half a century ago, it was shown that supraphysiological glucose fluctuations can induce renal lesions characteristic of diabetic nephropathy in rats [80]. Many years later, the biochemical aspects of this effect were identified. It was demonstrated that IHG (5–25 mmol/L) increases the production of collagen types I, III and IV in cultured mesangial cells, and type III collagen synthesis increases to a greater extent when stimulated by oscillatory glucose rather than CHG [81]. In proximal tubular cells, IHG was found to be a more potent stimulating factor for the secretion of transforming growth factor beta (TGF-β1), one of the most powerful fibrogenic mediators. In both cortical fibroblasts and proximal tubular cells, IHG increases collagen synthesis [82]. In cortical fibroblasts, fluctuating glucose enhances the production of collagen IV and fibronectin. In addition, it increases the synthesis of tissue inhibitor of matrix metalloproteinase, inhibiting matrix degradation. A short-term (90 min) increase in the glucose concentration stimulates TGF-β1 secretion by fibroblasts [83]. Thus, excessive glucose fluctuations can cause a more pronounced fibrogenic effect in a diabetic kidney than persistent hyperglycemia. This fact is consistent with data indicating that high GV is associated with a decline in renal function in diabetic rats [84] and patients with T2D [85,86].

### 2.7. Beta Cell Dysfunction

An inverse relationship between beta cell function and GV was observed in subjects with both T1D [87] and T2D [88,89]. Obviously, a compromised insulin response causes an increase in GV. At the same time, excessive GV can contribute to the progressive deterioration of beta cell function. It was found that IHG induces a more significant impairment of the glucose-stimulated insulin release response in rat islets and insulinoma cells (INS-1) than CHG, and this effect is related to the stress of the endoplasmic reticulum and oxidative stress [24]. When incubated under IHG conditions, INS-1 demonstrated a reduction in the response to glucagon-like peptide 1 [90]. In these cells, IHG generated a more toxic effect than CHG, including both apoptosis-inducing and antiproliferative activity [17]. A deteriorating effect of IHG on apoptosis and insulin release could be diminished by antioxidant pretreatment [91]. In rats, either continuous or intermittent hyperglycemia induced beta cell dysfunction and insulin resistance [25]. Chronic oscillating glucose caused beta cell dedifferentiation and failure in rats [67]. The long-term effect of enhanced GV on beta cell function and plasticity needs further research.

**Figure 1:**
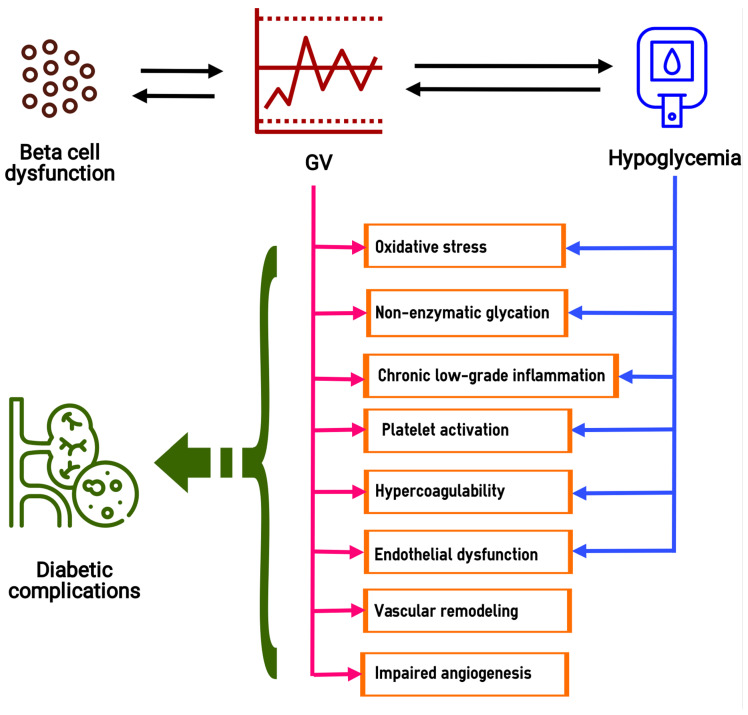
GV-related biochemical and pathophysiological abnormalities in the pathogenesis of diabetes complications. GV, glucose variability. The dotted lines in the GV block illustrate the excessive glucose oscillations. The green dashed arrow shows supposed relationships between pathophysiological processes, induced by high GV and hypoglycemia, and diabetic complications.

Thus, the role of supraphysiological glucose fluctuations in the pathogenesis of vascular complications of diabetes is realized through non-enzymatic glycation, oxidative stress, chronic low-grade inflammation, endothelial dysfunction, vascular remodeling, angiogenesis disorders, activation of blood cells (platelets and leukocytes), hypercoagulability and renal fibrosis (Figure 1). Some of these abnormalities are exacerbated by hypoglycemia, which is at an increased risk in patients with high GV. Finally, oscillating hyperglycemia contributes to beta cell dysfunction, which further increases GV and completes the vicious circle.

## 3. Cell Biology under High-GV Conditions

### 3.1. Altered Mitochondrial Homeostasis

Abnormalities of mitochondrial biogenesis, fission, fusion and mitophagy are reported to be involved in impaired oxidation, reduced mitochondrial contents and excessive ROS production in diabetes [92]. Specifically, the signs of altered mitochondrial homeostasis and mitochondrial dysfunction were observed in the diabetic kidney [93], retina [94], heart [95] and pancreatic beta cells [96].

Mitochondrial dysfunction could be considered a cornerstone in the development of GV-related oxidative stress. In cultured HUVECs, oscillating glucose induced ROS generation and an altered mitochondrial membrane potential [97]. Similar changes were recorded in INS-1 cells under IHG conditions [98]. In astroglial cells, up and down glucose fluctuations induced mitochondrial dysfunction, which was accompanied by oxidative/nitrosative stress, impaired glutamate metabolism and increased proinflammatory cytokine secretion [99]. In hepatic L02 cells incubated with palmitic acid, IHG induced more pronounced oxidative stress and mitochondrial dysfunction compared to CHG. Treatment with cyclosporin A, a mitochondrial permeability transition inhibitor, prevented mitochondrial dysfunction, oxidative stress and hepatocyte apoptosis in a model of high GV in high-fat diet C57BL/6J mice [100]. In a model of ischemia/reperfusion injury in the diabetic heart, glucose fluctuations increased the levels of miRNA-200c and miRNA-141. These changes were associated with decreased activities of mitochondrial superoxide dismutase and catalase and enhanced ROS production [101].

At present, little is known about the effect of GV on mitochondrial respiration and bioenergetics. It was demonstrated that in human islet cells, exposure to CHG for 4 days induced an increase in mitochondrial respiration and the cytosolicATP/ADP ratio [102]. Similarly, glucose fluctuations intensified aerobic glycolysis in cultured mouse mesangial cells. Oscillating glucose lowered the activity of aconitase, an enzyme of the Krebs cycle, and suppressed mitochondrial respiratory chain complex I [103]. At the same time, a reduction in the mitochondrial complex I activity was observed in the rat brain in a model of T1D [104]. Therefore, this effect may be induced by hyperglycemia per se, rather than increased GV. The role of glucose fluctuations in altering the mitochondrial respiratory chain requires further research.

### 3.2. Endoplasmic Reticulum Stress

The endoplasmic reticulum (ER) is considered to be important for nutrient sensing in many cell types, including hepatocytes, adipocytes, muscle cells, neurons and beta cells. An imbalance between the demand and capacity of the ER for protein folding is referred to as ER stress. Evidence is accumulating on the role of ER stress in the development of diabetes [105] and its complications, including retinopathy, nephropathy and neuropathy [106].

In diabetes, excessive glucose exposure alters ER homeostasis, and high GV may be an additional trigger for ER stress. It was demonstrated that in human retinal pericytes, IHG, but not CHG, increases the expression of activating transcription factor 4 (ATF4) and C/EBP homologous protein (CHOP), key mediators of ER stress-associated inflammation and cell death [107]. In cultured rat pericytes, strong unfolded protein response activation leading to apoptosis was observed when glucose was reduced from high to low levels, or the zero level [108]. High GV turned out to be a more powerful inductor of ER stress-related apoptosis compared with CHG in cultured rat vascular smooth muscle cells [67]. The modeling of recurrent short-term hypoglycemia and hyperglycemia induced apoptosis and oxidative stress via the response to ER stress in mouse Schwann cells [109]. In subjects with metabolic syndrome, the glucose load in the oral glucose tolerance test enhanced the expression of spliced XBP-1, Grp78 and calreticulin, the ER stress markers, in mononuclear cells. These changes were accompanied by a significant increase in the expression of inflammatory cytokines interleukin 1 α/β, IL-6 and interleukin 8 [110]. These data clearly indicate the role of glucose fluctuations in the generation of ER stress in diabetes.

### 3.3. Autophagy

Autophagy is a process of self-degradation and reconstruction of damaged organelles and proteins via lysosomes. This cellular recycling is vital for highly differentiated cells, including neurons, podocytes, cardiomyocytes, retinal cells and beta cells [111,112]. The impaired autophagy plays a role in the development of both T1D and T2D [113,114,115,116], and diabetic kidney disease [117,118,119].

Glucose seems to be a prominent regulator of autophagy [120,121,122]. Glucose levels indirectly affect autophagy in many cell types through the regulation of glucagon and insulin secretion. Glucagon is known as a potent stimulator of autophagy, whereas insulin suppresses it by stimulating mammalian target of rapamycin complex 1 (mTORC1) [122]. In cells, glucose withdrawal causes ATP depletion, which stimulates AMP-activated protein kinase (AMPK) and the AMPK–S-phase kinase-associated protein 2 (SKP2)–coactivator-associated arginine methyltransferase 1 (CARM1) signaling pathway, an upstream activator of autophagy [122,123,124]. On the other hand, impaired autophagy can influence insulin sensitivity through the changes in glucose transporter type 4 (GLUT4) degradation and recovery [125]. Moreover, impaired glycophagy, a selective autophagy in the liver, heart and muscles, could contribute to hyperglycemia [126].

It was shown that IHG causes enhancement of the autophagic flux in cultured HUVECs [74] and rat podocytes [22]. Similarly, in human retinal pigmented epithelial cells, IHG significantly increased the generation of autophagosomes, decreased the expression of an autophagy receptor, p62, a marker of suppressed autophagy, and induced the conversion of an autophagosome-associated protein microtubule-associated protein 1A/1B light chain 3B (LC3) I to its active form LC3 II [127]. The role of GV in altering autophagy in vivo requires further research.

### 3.4. Apoptosis

The metabolic changes and dysfunction of organelles under high-GV conditions ultimately reduce the survival of a number of cells. Some in vitro studies have demonstrated the pro-apoptotic effect of IHG in endothelial cells [25,128,129], mesangial cells [103,130], cardiomyocytes [131], neurons [132,133], glial cells [134], Schwann cells [135] and beta cells [98,136]. The activation of GV-related apoptosis was attributed to mitochondrial dysfunction, ER stress and autophagy [100,124,127,131].

Glucose fluctuations have been validated to be more harmful than CHG in exacerbating the apoptosis of beta cells. In cultured INS-1, IHG induced apoptosis by the significant up-regulation of pro-apoptotic proteins caspase-3 and 9, and by down-regulation of the antiapoptotic protein Bcl-2 [98].

Wu N. et al. performed in vivo experiments demonstrating the effect of acute glucose fluctuations on the levels of apoptosis regulators in aorta endothelial cells in rats. Animals with glucose fluctuations induced by intermittent glucose infusions demonstrated reduced Bcl-2 and pro-caspase-3 levels, and enhanced Bax mitochondrial translocation and caspase-3 p17 protein levels, in comparison with those with persistent hyperglycemia [25]. In the high-GV model established by insulin and glucose injections in rats with diet- and streptozotocin-induced diabetes, the predominance of pro-apoptotic regulators with an increased Bax/Bcl-2 ratio was found [84]. Interestingly, sodium-glucose cotransporter 1 (SGLT1) knockdown down-regulated Bax expression, up-regulated Bcl-2 expression and reduced caspase-3 activation induced by high GV in cultured rat H9c2 cardiomyocytes [131].

The activation of apoptosis is among the most important mechanisms of neurodegeneration in diabetes. It was shown that IHG induces the oxidative stress-related apoptosis of Schwann cells by both caspase-dependent and caspase-independent pathways. The cytotoxic effect of IHG was significantly more potent than that of CHG [14,15]. The central nervous system, being highly dependent on the glucose supply, becomes especially vulnerable in conditions of high GV. In diabetic rats, intermittent hyperglycemia turned out to be a more critical factor, promoting neuron apoptosis and inducing inflammation in the hippocampus, than CHG [132]. At the same time, acute glucose fluctuations affect microglial activity. It was demonstrated that a sharp increase in the glucose level (from 5.5 to 25 mmol/L) promotes cell growth, induces oxidative and inflammatory stress and activates microglial cells. The reverse shift from hyperglycemia to normoglycemia trapped microglia in a state of metabolic stress, which triggered apoptosis and autophagy [134].

### 3.5. Cell Proliferation

Enhanced GV can modulate the proliferative response. It was found that either CHG or IHG induces the proliferation of vascular smooth muscle cells (VSMCs) in vitro [137]. Fluctuating glucose increased the proliferation and migration of VSMCs in an OLETF rat T2D model [138]. Earlier research demonstrated that IHG enhances cell proliferation and VEGF expression in retinal endothelial cells. These changes were associated with ROS overproduction at the mitochondrial transport chain [21].

At the same time, GV can suppress the proliferation of endothelial cells, podocytes and beta cells. Both IHG and CHG decreased the proliferation of cultured HUVECs [74]. It was revealed in INS-1 culture that IHG decreases beta cell viability and induces G0/G1 cell cycle arrest. INS-1 demonstrated a decreased expression of mitogen factors cyclin D1 and S-phase kinase-associated protein 2, whereas the expression of cyclin-dependent kinase inhibitors 1A and 1B, two antiproliferative factors, was increased [17,139].

**Figure 2:**
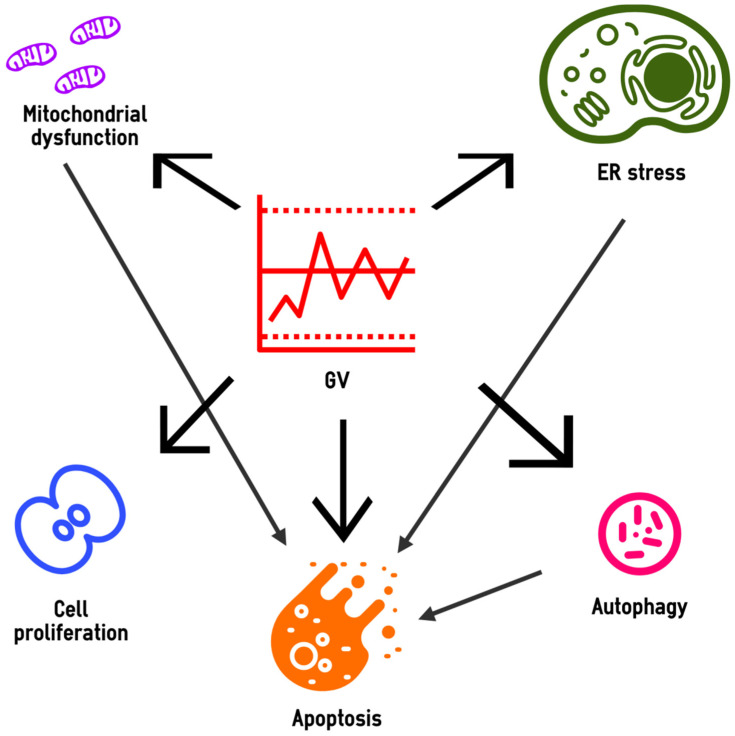
Cellular events promoting the high GV effects on target organs in diabetes. GV, glucose variability; ER, endoplasmic reticulum. The dotted lines in the GV block illustrate the high glucose excursions. The thick arrows show the cellular events induced by high GV, the thin arrows demonstrate the relationships between these events.

Thus, high GV can promote many events in the targeted cells, including mitochondrial dysfunction, ER stress, changes in the intensity of autophagic flux, apoptosis activation and abnormalities in the proliferative response (Figure 2).

## 4. Molecular Mechanisms of the High GV Effects in the Target Cells

### 4.1. Gene Expression

Although there are scarce data on changes in gene expression induced by excessive GV, there is a large pool of studies on gene profiling related to hyperglycemia. Using high-throughput technologies, differential gene expression was measured under hyperglycemic conditions in beta cells [140,141], pancreatic cells [142], hepatic cells [143,144], endothelial cells [145], myotubes [146], cardiomyocytes [147], vascular smooth muscle cells [148,149], adipose progenitor cells [150], kidney cells [151], renal tubular epithelial cells [152], retina [153,154], immune cells [155,156] and others. The genes that demonstrate an altered expression in hyperglycemia are mostly involved in glucose metabolism, inflammation and immune processes, endothelial dysfunction, angiogenesis, oxidative stress, mitochondrial dysfunction, hypoxia and cell death.

Transcriptomic studies have revealed the effect of hyperglycemia on the expression profile of a large number of genes. More than 80 genes involved in hepatic lipid metabolism were differentially expressed in hyperglycemic rats with a model of T1D [143]. With the use of high-throughput RNA sequencing, it was demonstrated that hyperglycemia has a strong effect on HepG2 cells, with 4259 genes showing a differential expression. These genes participate in cholesterol metabolism, DNA replication, complement and clotting cascades [144]. Maier et al. hypothesized that hyperglycemia amplified the expression of genes induced by thrombospondin-1 in vascular smooth muscle cells. Microarray analysis revealed that hyperglycemia altered the expression of 30 genes, while hyperglycemia combined with thrombospondin-1 altered the expression of 2822 genes. These findings suggest that hyperglycemia may significantly enhance the thrombospondin-1 effect on atherosclerosis progression [148].

Fewer studies have focused on gene expression in hypoglycemia. In sirtuin 6-deficient mice that developed a lethal early-life hypoglycemia, the microarray revealed nearly 200 genes with an altered expression. These genes were involved in glucose metabolism, nutrient stress responses, glycolysis and mitochondrial function [157]. A gene response to insulin-induced hypoglycemia was estimated in the mouse retina by an array. Genes whose expression was modified by low glucose were enriched in lysosomal function, glutathione metabolism and apoptotic pathways and potentially involved in retinal cell death [158]. A set of genes specifically activated by recurrent hypoglycemia was revealed in a study of whole genome expression profiling after recurrent hypoglycemia and acute hypoglycemia in the adrenal medulla of normal Sprague Dawley rats. These genes were related to the activation of the unfolded protein response, impaired epinephrine secretion, increased neuropeptide signaling, altered ion homeostasis and down-regulation of genes involved in Ca^2+^-dependent exocytosis of secretory vesicles [159].

It was found that even short-term enhanced GV could adversely affect gene expression in the arterial wall. In the study of Zervou et al., pIns-c-MycER(TaM) transgenic mice were successively exposed to hypo- and hyperglycemia, after which they recovered for up to 3 months. The expression of 95 genes was significantly affected by hypoglycemia, and 769 genes were affected by hyperglycemia. These genes were involved in atherogenic processes, including inflammation and arterial calcification. Although the expression of many genes returned to its initial level after 3 months, in one in four genes, recovery was not observed [160]. These data indicate that non-physiological glucose fluctuations may have a prolonged effect on gene expression. Further research in this direction is urgently needed.

Recently, we performed the bioinformatic reconstruction and analysis of the gene network of GV. The network consisted of 37 genes/proteins associated with both hyperglycemia and hypoglycemia. GV-related molecules were involved in glucose metabolism, intracellular signaling, cell proliferation and other biochemical/physiological processes; they were identified in the central positions of the gene networks of diabetic vascular complications [161].

### 4.2. Epigenetic Modifications

Glucose can induce a number of epigenetic modifications that significantly alter the functioning and vital activity of various cell types. In a pivotal work in this field, El-Osta et al. demonstrated that transient elevation in the glucose level causes long-lasting epigenetic changes in the NF-κB subunit p65 promoter in aortic endothelial cells in vitro and in non-diabetic mice. These changes were associated with an increased p65 gene expression that persisted for at least 6 days of subsequent normal glucose levels, and NF-κB-induced increases in MCP-1 and VCAM-1 expression [162]. These data clearly indicate that epigenetic modifications may be an important mechanism in GV-induced vascular inflammation and dysfunction.

Costantino S. et al. found DNA hypomethylation and histone 3 acetylation on the p66^Shc^ promoter of the SHC-transforming protein 1 gene *(SHC1)*, resulting in gene overexpression, in patients with T2D. CGM-derived MAGE and postprandial glucose, but not HbA1c, were associated with the epigenetic profile. The intensification of glycemic control over 6 months did not eliminate the changes [163]. The mechanism of p66^Shc^-reduced CpG methylation could be related to methyltransferase DNA (cytosine-5-)-methyltransferase 3 beta (DNMT3b), an enzyme playing an important role in the maintenance of DNA methylation. Sirtuin 1 (SIRT1) could be involved in H3 deacetylation of p66^Shc^ [163,164]. In patients with T2D, the expression of DNMT3b and SIRT1 was inhibited compared to the control [163].

Recently, the effect of glucose on whole genome DNA methylation was studied in human retinal endothelial cells and HUVECs [165]. The lines were exposed to basal (5 mmol/L) or high (25 mmol/L) glucose-containing media for variable lengths of time. When comparing the endothelial cells, incubated for 2 days versus 7 days, 17,354 and 128 differentially methylated CpGs in 88 and 8 differentially methylated regions were identified for HUVECs and retinal endothelial cells, respectively. Pathway enrichment analyses highlighted the involvement of regulators of embryonic development (i.e., HOX genes), TGF-β signaling, bone morphogenetic protein (BMP) signaling, runt-related transcription factor 2 (RUNX2) transcriptional regulation and the complement cascade.

It was demonstrated that fluctuating glucose significantly decreased the phosphorylation of the endothelial nitric oxide synthase (eNOS) at Ser-1177 and increased the phosphorylation of JNK and p38, leading to the damage of vascular endothelial cells [166]. IHG lowered the phosphorylation levels of protein kinase B (v-akt murine thymoma viral oncogene homologue, Akt), AMPK and glycogen synthase kinase 3 beta (GSK3β), influencing the function of endothelial cells and beta cells [19,91,167].

Small single-stranded non-coding RNAs (miRNAs) have been discussed as another method of epigenetic regulation. Aberrant miRNA expression is implicated in the pathogenesis of numerous diseases, including diabetes and its complications [168]. In HUVECs cultured under IHG conditions, 13 miRNAs were differentially expressed. miR-1273g-3p partially mediated the effect of IHG on the autophagy, migration and proliferation of HUVECs [74]. Another example of GV-induced miRNA-dependent changes is a phenotype polarization switch of microglia. In microglial cells, glucose fluctuations induce polarization transitions from M2 to M1. The M1 phenotype has proinflammatory effects and can be responsible for neuronal damage; in contrast, M2-polarized microglia can inhibit the inflammatory response and promote nerve repair. It was found that miR-124, miR-145, miR-146a and miR-711 are implicated in the M2 phenotype polarization of microglia, while miR-689 and miR-155 are involved in M1 polarization. In macrophages, miR-124 and miR-146a induced M2 phenotype polarization [169]. In the glucose fluctuation cell model, miR-129-3p suppressed glucose-mediated hippocampal neuronal damage. Specifically, miR-129-3p overexpression produced a dramatic reduction in calcium overload, ROS generation and an increase in antioxidant activity [170]. In cultured HUVECs, miR-1273g-3p mediates the effect of GV on autophagy and endothelial dysfunction [74]. In human endothelial cells, miR-185 and miR-21 were induced by oscillating glucose, leading to an impaired antioxidant response by the dysregulation of glutathione peroxidase 1 and superoxide dismutase 2 [97,171]. It was demonstrated that IHG induces the up-regulation of HIF-1α and miR-210 in glomerular mesangial cells, which might play a pivotal role in the series of molecular events triggered by GV [22].

Thus, the effects of glucose fluctuations on gene expression can be exacerbated and prolonged by epigenetic modifications. At present, glucose-induced epigenetic modifications and related changes in the activity of signaling pathways are considered as an important mechanism of “metabolic memory” or “metabolic karma” in diabetes [168,172,173].

### 4.3. Signaling Pathways

The cellular and molecular effects of GV are realized through a variety of signaling pathways. The activation of PKC is among the initial molecular events under high-glucose conditions. PKC is a driver of numerous signal transduction cascades that regulate cell metabolism and plasticity. Among the downstream targets of PKC is NADPH oxidase that activates superoxide production and thus exacerbates oxidative damage [174].

A number of molecular effects of oxidative stress are mediated via NF-κB-dependent signaling pathways. NF-κB is a universal transcription factor that controls the expression of genes for the immune response, apoptosis and cell cycle. In diabetes, ROS, AGEs and angiotensin II induce an inflammatory response, endothelial dysfunction and renal fibrosis via the activation of NF-κB. Accordingly, NF-κB is considered as a potential target in diabetic vascular complications [175]. As it has previously been mentioned, transient high glucose induces prolonged NF-κB activation in vascular endothelial cells [162]. The IHG-stimulated activation of NF-κB in cultured HUVECs down-regulated the expression of bcl-2, an antiapoptotic protein [176]. In vascular cells, glucose fluctuations promote the dysfunction of large-conductance, calcium-activated potassium channels via the overproduction of ROS and activation of PKCα/NF-κB/MuRF1 signaling [177]. ROS-mediated NF-κB activation under high-GV conditions up-regulates the receptor for AGEs in podocytes [22].

The dysregulation of the phosphoinositide-3-kinase (PI3K)/Akt, mitogen-activated protein kinase (MAPK) and AMPK pathways is considered to be involved in altered glucose metabolism and related biochemical abnormalities in diabetes and high GV [178,179]. The PI3K/Akt signaling pathway, which is essential for cell survival and growth, plays an important role in preventing endothelial cell injury induced by high glucose. It was shown that IHG induces a more severe decrease in the phosphorylation of Akt and GSK-3β than CHG in cultured HUVECs; this effect is associated with reduced cell viability [19]. In agreement with these data, it was demonstrated that IHG suppresses NO synthesis in cultured HUVECs to a greater extent than CHG via the inhibition of PI3K/Akt and eNOS activity [58]. The pro-apoptotic effect of IHG in cultured neuronal cells (PC12 cell line) also involves the PI3K/Akt pathway [133]. The oxidative and inflammatory stress and microglial activation induced by acute glucose fluctuation in the mouse microglial BV-2 cell line were mediated through the PI3K/Akt, NF-κB and MAPK cascades [134].

MAPK families play an important role in cell proliferation, differentiation and apoptosis. The MAPK families include extracellular signal-regulated kinase (ERK), JNK and p38 MAPK [180]. Some data point to the role of these signaling molecules in the realization of the effects of GV in the target organs. It was demonstrated that MAPK (ERK1/2), as well as the PI3K and NF-κB signaling pathways, is involved in the proliferative effect of IHG in VSMCs [138].

In vascular endothelial cells, IHG increased the phosphorylation of JNK [166]. The JNK pathway plays a central role in the cell response to hyperglycemia, oxidative stress, proinflammatory cytokines and other stress-inducing stimuli. The JNK-dependent effects include the regulation of gene expression, cell death and cellular senescence [181,182]. In patients with diabetes, JNK contributes to vascular insulin resistance and endothelial dysfunction [183]. It was demonstrated both in vivo and in vitro that the PKC/JNK pathway mediates the pro-apoptotic effect of glucose fluctuations in endothelial cells [129,184].

In diabetes, high glucose activates p38 MAPK signaling [181]; high GV may be an additional trigger of the event [166]. It was shown that GV generates the more severe up-regulation of type I collagen synthesis and fibrosis of aorta via the activation of the ROS/p38 MAPK/Runx2 pathway in Sprague Dawley rats with streptozotocin-induced diabetes [185]. In astroglial cells, glucose fluctuations induce toxicity with oxidative and inflammatory stress by activating p38 MAPK and NF-κB [99].

The interactions among the MAPK, NF-κB and TGF-β/Smad signaling pathways are essential for fibrogenesis. It is well known that TGF-β’s biological effects were realized by activating downstream mediators Smad2 and Smad3, which is negatively regulated by an inhibitory Smad7 [186]. The activation of the MAPK/ERK and TGF-β/Smad signaling pathways is considered as a cornerstone in the pathogenesis of renal fibrosis in diabetic kidney disease [187]. As it was demonstrated in mice with alloxan diabetes, excessive blood glucose fluctuations cause the more pronounced activation of the TGF-β/Smad2 and ERK/MAPK pathways in the kidney compared to stable hyperglycemia. These changes in signal transduction were accompanied by the marked increase in type I collagen synthesis and suppression of collagen degradation [188]. The inhibition of skin collagen synthesis and increase in collagen degradation under high GV is also attributed to both the MAPK and Smad signaling pathways [189].

AMPK is a master regulator of metabolism which acts as an intracellular sensor of energy availability [178]. The glucose shortage promotes AMPK activity; meanwhile, overnutrition inhibits it. In many cell types, AMPK stimulates glucose uptake via trafficking glucose transporters GLUT1 and GLUT4; acutely stimulates glycolysis; and, in the longer term, tends to promote oxidative metabolism. The activation of autophagic flux via ULK1 is considered as an important AMPK-dependent mechanism of cellular metabolic adaptation [190,191]. Recently, it has been demonstrated that high glucose represses AMPK signaling via MG53 E3 ubiquitin ligase-mediated AMPKα degradation and deactivation [192]. Currently, little is known about the effect of GV, which is characterized by intermittent glucose excess and deprivation, on AMPK activity in diabetes. It was found that the activation of AMPK by globular adiponectin can inhibit, at least partially, the IHG-induced apoptosis of HUVECs [193].

mTORC1 is another protein kinase that is regulated by glucose availability; however, unlike AMPK, mTORC1 is activated in high-glucose conditions. When it is activated, mTORC1 shifts the metabolic paradigm towards anabolic processes, promotes cell growth and suppresses autophagic flux [191]. It was demonstrated that the inhibition of AMPK by high glucose inversely correlates with the activation of the mechanistic target of rapamycin (mTOR) pathway in beta cells [194]. It is currently known that upon glucose depletion, mTORC1 is inhibited by AMPK-dependent and AMPK-independent mechanisms [195]. Recent data indicate that aldolase could be a sensor for both low and high glucose levels, linking to the AMPK and mTORC1 pathways [196]. In cancer, diabetes and other diseases characterized by abnormal glucose metabolism, mTORC1 is deregulated [195,197]. In diabetes, hyperactivated mTORC1 is involved in the pathogenesis of cardiomyopathy [198], diabetic retinopathy [199] and diabetic kidney disease [200]. Unfortunately, the role of mTORC1 signaling in GV-related vascular effects has not been studied to date.

Thus, the deteriorative effects of high GV in the target cells are realized through the PI3K/Akt, NF-κB, MAPK (ERK), JNK, TGF-β/Smad and other signaling pathways (Table 1). Elucidating the pathophysiological role of AMPK and mTORC1 under fluctuating glucose conditions is a promising challenge for future research.

## 5. Conclusions

Current data indicate that the deteriorating effect of high GV on the targeted cells may be realized through a number of molecular abnormalities. Fluctuations in glucose levels alter the expression profile of a large number of genes and modulate the activity of intracellular signaling pathways. Epigenetic modifications prolong the effects of GV. These changes cause the dysfunction of cell organelles and disrupt the life cycle and synthetic function of endothelial cells and other cells of the vascular wall, the nervous system, the kidneys, the liver and other organs. These changes are manifested by biochemical and pathophysiological abnormalities underlying diabetic complications. The multiple deteriorative effects of GV provide further support for considering it as a therapeutic target in diabetes. Treatment modalities focused on reducing GV may have an advantage in diabetes management. Further study of the cellular and molecular effects of high GV is needed to develop targeted methods for the treatment and prevention of diabetic vascular and neural complications.

## Figures and Tables

**Table 1:** The principal signaling pathways mediating the pathophysiological effects of high GV in diabetic complications.

Effect	Pathways	Refs.
Oxidative stress in endothelial and neural cells	PKC/NF-κB, PI3K/Akt, p38MAPK	[99,134,174,175]
Endothelial dysfunction and apoptosis	PI3K/Akt, NF-κB, PKC/JNK	[19,58,129,176,184]
Proliferation of VSMCs	MAPK (ERK1/2), PI3K/Akt, NF-κB	[138]
Vascular low-grade inflammation	NF-κB and p38 MAPK	[162]
Renal fibrosis	MAPK (ERK1/2) and TGF- β/Smad	[188]
Aortic fibrosis	TGF-β/Smad, NF-κB, p38 MAPK and Runx2	[185]
Neuronal apoptosis and neurodegeneration	PI3K/Akt, NF-κB	[133,134]

## Data Availability

The data presented in this study are available on request from the corresponding author.

## Abbreviations

AGEs	Advanced glycation end-products
AMPK	AMP-activated protein kinase
ATF4	Activating transcription factor 4
BMP	Bone morphogenetic protein
CARM1	Coactivator-associated arginine methyltransferase 1
CGM	Continuous glucose monitoring
CHOP	C/EBP homologous protein
CHG	Constantly high glucose
DNMT3b	DNA (cytosine-5-)-methyltransferase 3 beta
eNOS	Endothelial nitric oxide synthase
EPCs	Endothelial progenitor cells
ER	Endoplasmic reticulum
ERK	Extracellular signal-regulated kinase
GLUT4	Glucose transporter type 4
GSK3β	Glycogen synthase kinase 3 beta
GV	Glucose variability
HbA1c	Glycated hemoglobin A1c
hsCRP	High-sensitivity C-reactive protein
HMGB1	High-mobility group box 1
HUVECs	Human umbilical vein endothelial cells
ICAM-1	Intercellular adhesion molecules 1
IHG	Intermittently high glucose
INS-1	Insulinoma cells
IRF5	Interferon regulatory factor 5
JNK	c-Jun N-terminal kinase
LAMP	Lysosomal-associated membrane protein
LC3	Microtubule-associated proteins 1A/1B light chain 3B
MAGE	Mean amplitude of glucose excursions
MAPK	Mitogen-activated protein kinase
MCP-1	Monocyte chemoattractant protein 1
miRNAs	small single-stranded non-coding RNAs
mTOR	Mechanistic target of rapamycin
mTORC1	Mammalian target of rapamycin complex 1
NADPH	Nicotinamide adenine dinucleotide phosphate
NF-κB	Nuclear factor kB
PAI-1	Plasminogen activator inhibitor-1
PI3K	Phosphoinositide-3-kinase
PKC	Protein kinase C
ROS	Reactive oxygen species
RUNX2	Runt-related transcription factor 2
SHC1	SHC-transforming protein 1
SKP2	S-phase kinase-associated protein 2
SD	Standard deviation
sICAM-1	Soluble intercellular adhesion molecules 1
SIRT1	Sirtuin 1
SGLT1	Sodium-glucose cotransporter 1
SOD2	Superoxide dismutase 2
T1D	Type 1 diabetes
T2D	Type 2 diabetes
TGF-β1	Transforming growth factor beta 1
TLR4	Toll-like receptor 4
TNF-α	Tumor necrosis factor α
TSP-1	Thrombospondin-1
TUNEL	Terminal deoxynucleotidyl transferase dUTP nick end labeling
VCAM-1	Vascular cell adhesion molecules 1
VEGF	Vascular endothelial growth factor
VSMCs	Vascular smooth muscle cells
